# Assessing combining abilities, genomic data, and genotype × environment interactions to predict hybrid grain sorghum performance

**DOI:** 10.1002/tpg2.20127

**Published:** 2021-08-09

**Authors:** Jales M. O. Fonseca, Patricia E. Klein, Jose Crossa, Angela Pacheco, Paulino Perez‐Rodriguez, Perumal Ramasamy, Robert Klein, William L Rooney

**Affiliations:** ^1^ Dep. of Soil and Crop Sciences Texas A&M Univ. College Station TX 77843 USA; ^2^ Dep. of Horticultural Sciences Texas A&M Univ. College Station TX 77843 USA; ^3^ International Maize and Wheat Improvement Center (CIMMYT) Él Batán Mexico; ^4^ Colegio de Postgraduados Montecillos Edo. de Mexico Texcoco Mexico; ^5^ Agriculture Research Center Kansas State Univ. Hays KS 67601 USA; ^6^ Southern Plains Agricultural Research Center USDA‐ARS College Station TX 77845 USA

## Abstract

Genomic selection in maize (*Zea mays* L.) has been one factor that has increased the rate of genetic gain when compared with other cereals. However, the technological foundations in maize also exist in other cereal crops that would allow prediction of hybrid performance based on general (GCA) and specific (SCA) combining abilities applied through genomic‐enabled prediction models. Further, the incorporation of genotype × environment (G × E) interaction effects present an opportunity to deploy hybrids to targeted environments. To test these concepts, a factorial mating design of elite yet divergent grain sorghum lines generated hybrids for evaluation. Inbred parents were genotyped, and markers were used to assess population structure and develop the genomic relationship matrix (GRM). Grain yield, height, and days to anthesis were collected for hybrids in replicated trials, and best linear unbiased estimates were used to train classical GCA‐SCA–based and genomic (GB) models under a hierarchical Bayesian framework. To incorporate population structure, GB was fitted using the GRM of both parents and hybrids. For GB models, G × E interaction effects were included by the Hadamard product between GRM and environments. A leave‐one‐out cross‐validation scheme was used to study the prediction capacity of models. Classical and genomic models effectively predicted hybrid performance and prediction accuracy increased by including genomic data. Genomic models effectively partitioned the variation due to GCA, SCA, and their interaction with the environment. A strategy to implement genomic selection for hybrid sorghum [*Sorghum bicolor* (L.) Moench] breeding is presented herein.

## Core Ideas

- Genomic and classical GCA‐SCA–based models can predict agronomic traits in sorghum hybrids.
- Genomic models that include G × E effects can increase the prediction accuracy of sorghum hybrids.
- Genomic models can incorporate the natural population structure existent in hybrid crops.
- Hybrids adapted to a target environment provide better predictions than non‐adapted hybrids.

**Abbreviations**
BLUE: best linear unbiased estimate
BLUP: best linear unbiased predictor
BRR: Bayesian Ridge Regression model
BV: breeding value
CI: coincidence index
CV: cross‐validation
CVe: coefficient of variation
GB: genomic Bayesian model
GBLUP: genomic BLUP
GBS: genotyping‐by‐sequencing
GCA: general combining ability
GRM: genomic relationship matrix
G × E: genotype × environment
MAF: minor allele frequency
MM: multi‐environment main genetic effect model
MMGE: multi‐environment main genetic effect model plus
PCoA: principal coordinate analysis
*r*
: prediction accuracy
REML: restricted maximum likelihood
SCA: specific combining ability
SI: selection intensity
SM: single‐environment main genetic effect model
SNP: single nucleotide polymorphism

## 1 INTRODUCTION

Genotypic data in breeding programs evolved from mapping simple inherited characteristics to the use of genetic markers for detailed genome‐wide association studies of quantitatively inherited traits (Nadeem et al., 2018). Despite these advancements, the successful application of marker‐based genomic technologies for crop improvement is limited (Jannink et al., 2010). However, with the advent of genomic selection (GS), a growing wealth of information indicates that genome sequencing technologies are a valuable resource to augment traditional phenotypic‐based crop improvement efforts. A method for improving quantitative traits, GS relies on genomic‐enabled prediction models to predict genetic values using all available molecular markers across the genome. A seminal report by Meuwissen et al. (2001) demonstrated the principles of GS by predicting breeding values (BV) via simulation studies, and the authors proposed that the implementation of GS in animal and plant breeding programs could increase the rate of genetic gain.

In crop improvement, genomic prediction studies in several crops have shown the potential to increase rates of genetic gain over classical phenotypic‐based methodologies (Crossa et al., 2014; He et al., 2016; Bernal‐Vasquez et al., 2017; Tan et al., 2017; Dias et al., 2018; Hunt et al., 2018; Xu et al., 2018; Islam et al., 2020). Additionally, more cost‐effective genotyping techniques have encouraged plant breeders to develop suitable models to introduce GS into the breeding pipeline (Eathington et al., 2007; Heffner et al., 2009, 2010; Bhat et al., 2016). Although these GS methods have the potential to revolutionize the improvement in several crops, most of these methods have primarily benefitted a few crops that cover vast areas or have notably high value, including maize (*Zea mays* L.) and soybean [*Glycine max* (L.) Merr.] (Technow et al., 2012, 2014; Jarquín et al., 2014b; Stewart‐Brown et al., 2019). Other crops are beginning to experiment with GS in the breeding pipeline, but few studies have addressed the issues related to genotype × environment (G × E) interactions, population structure in elite germplasm, and their effects on effectively predicting hybrid performance. Consequently, more complex genomic prediction models may be required to account for G × E effects and include alternative prediction objectives, i.e., general combining ability (GCA) or hybrid performance (Piepho et al., 2008).

Early genomic prediction models included statistical assumptions that were not applicable to hybrid crops (Technow et al., 2012; de los Campos et al., 2015). For instance, standard genomic best linear unbiased prediction (GBLUP) models assume equal variance across all loci and population homogeneity (Clark et al., 2011). By their very nature, hybrid crops are bred with population structure (i.e., heterotic groups) and, consequently, less restrictive genomic prediction models for hybrid crops are needed (de los Campos et al., 2015). To circumvent these limitations, extensions of standard GBLUP models have been developed under a hierarchical Bayesian framework (Meuwissen et al., 2001; Clark et al., 2011; Heslot et al., 2012; Technow et al., 2012; Zhao et al., 2013; de los Campos et al., 2015; Ramstein & Casler, 2019). This approach models the genetic variation using prior information to define hyperparameters from which variances can be sampled to adjust the effect of each locus separately (Meuwissen et al., 2001; de los Campos et al., 2009; Gianola et al., 2009; Habier et al., 2011). Alves et al. (2019) showed some advantages of Bayesian models to predict the performance of maize hybrids.

**Core Ideas**

- Genomic and classical GCA‐SCA–based models can predict agronomic traits in sorghum hybrids.
- Genomic models that include G × E effects can increase the prediction accuracy of sorghum hybrids.
- Genomic models can incorporate the natural population structure existent in hybrid crops.
- Hybrids adapted to a target environment provide better predictions than non‐adapted hybrids.

Parametric Bayesian models can incorporate population structure through kernel methods that represent genetic similarities between inbred lines via a genomic (co)variance matrix (VanRaden, 2008; de los Campos et al., 2009). Essentially, kernels include dense whole‐genome marker information in genomic‐enabled prediction models via a genomic relationship matrix (GRM) whose dimensions are equal to the number of inbred lines. This drastically reduces problems related to data dimensionality (i.e., big P small n) (Gianola & de los Campos, 2008). Non‐linear kernels can also be used in semi‐parametric models, and several studies report superior accuracy on genomic predictions (Gianola & de los Campos, 2008; Crossa et al., 2011; Morota & Gianola, 2014; Costa‐Neto et al., 2020). The application of kernel methods in genomic prediction models substantially increased the opportunities for plant breeders. For example, kernels can be used with almost any information set (e.g., covariates, strings, images, and graphs) and, due to their flexibility, kernels can accommodate multiple information within a genomic prediction model (de los Campos et al., 2009; de los Campos et al., 2010; Crossa et al., 2011). Moreover, G × E effects can be designed with a kernel based on the interaction effect between markers (i.e., G‐matrix) and environments (Burgueño et al., 2012; Lopez‐Cruz et al., 2015; Cuevas et al., 2016, 2017; Acosta‐Pech et al., 2017; Sukumaran et al., 2018).

The GS methodologies developed for maize may be applicable to other hybrid grain crops such as sorghum [*Sorghum bicolor* (L.) Moench] (Hunt et al., 2018), rice (*Oryza sativa* L.) (Xu et al., 2014; Wang et al., 2017), sunflower (*Helianthus annuus* L.) (Mangin et al., 2017), and wheat (*Triticum aestivum* L.) (Basnet et al., 2019). Initial studies on genomic prediction models in sorghum indicated that GS could be effective (de Oliveira et al., 2018; Fernandes et al., 2018; Hunt et al., 2018; Velazco et al., 2019; dos Santos et al., 2020; Sapkota et al., 2020). However, GS is not implemented in most sorghum breeding pipelines due to limited model testing and optimization for predicting hybrid performance. For instance, genomic prediction studies in sorghum have focused on predicting BV (de Oliveira et al., 2018; Fernandes et al., 2018; Hunt et al., 2018; dos Santos et al., 2020). As such, these models predict only the GCA of untested sorghum inbred lines. In addition, most genomic prediction studies involving sorghum consist of diverse germplasm panels and/or testcrosses derived therefrom, but these populations are not representative of the heterotic groups used in hybrid seed production (Fristche‐Neto et al., 2018). Finally, given the importance of G × E effects in all crop improvement efforts and the extreme variability of sorghum production environments, the inclusion of G × E effects in genomic prediction models seems imperative to improve selection efficiency and hybrid deployment (Hunt et al., 2018).

This study introduces the concept of predicting the performance of hybrid grain crops using sorghum as a model species. We implement models with GCA and specific combining ability (SCA) genetic main effects in two forms, a classical and a genomic hierarchical Bayesian framework. Through this research, we have addressed critical issues associated with the implementation of GS into an applied hybrid crop improvement program including G × E interactions, population structure, environment‐specific training sets, and selection intensity on the coincidence between observed and predicted values. Based on the results, we propose a conceptual framework for implementing GS into the breeding pipeline for those hybrid crops that lack the level of resources employed in crops such as maize, which includes grain sorghum.

## 2 MATERIAL AND METHODS

### 2.1 Phenotypic data

A set of 20 grain sorghum inbred lines developed in sorghum breeding programs at Texas A&M AgriLife Research (College Station, TX) and Kansas State Research Center (Hays, KS) were selected and crossed using a factorial mating scheme (Comstock & Robinson, 1952). Each breeding program provided 10 lines equally represented by female (A‐lines) and male (R‐lines) parents that are adapted to their respective target environments and had been evaluated in hybrid combinations prior to this study (Supplemental Table S1). The 100 hybrids naturally divided into four groups of 25 hybrids: TAM (A)/TAM (R) (T × T), TAM (A)/KSU (R) (T × K), KSU (A)/TAM (R) (K × T), and KSU (A)/KSU (R) (K × K).

For phenotyping, hybrids were planted in a randomized complete block design (RCBD) with a set (i.e., the four groups of 25 hybrid combinations aforementioned) in replication adjustment. In each replication, randomization occurred within and between sets to keep the set structure; entries were randomized within the set while sets were randomly ordered. In 2018, the tests were in five environments: Monte Alto (RF) (26°21′06.1″ N, 97°53′50.3″ W), Victoria (VC) (28°47′24.4″ N, 96°50′22.6″ W), and College Station (CS) (30°32′56.6″ N, 96°26′11.5″ W) in Texas, and Garden City (GC) (37°59′21.4″ N, 100°48′52.5″ W), and Colby (COL) (39°22′56.6″ N, 101°04′45.0″ W) in Kansas. In 2019, the environments were similar except that the Monte Alto location was replaced by Taft (TA) (28°00′05.4″ N, 97°15′12.4″ W), Texas, and Garden City was replaced by Hays (HAY) (38°51′10.8″ N, 99°20′24.6″ W) in Kansas. These locations represent distinct adaptation zones and, in each test, agronomic practices standard to the location were implemented (Supplemental Table S2). In 2018 trials, each location had three replications whereas two replications were planted in each site in 2019. A plot consisted of two rows, approximately 5.3 m in length, with row spacing between 0.76 and 1.0 m, consistent with production practices in each environment.

In this study, three agronomic traits were measured. Days to anthesis (DA) were recorded as the number of days from planting to when 50% of the plants in the plot were at mid‐anthesis; at Kansas locations, DA was collected only on the first replication. Just prior to harvest, plant height (PHT) was measured (in cm) using a representative plant in each plot at the length from the soil to the tip of the panicle. Plots were harvested using plot combines fitted with a Harvest Master GrainGage System (Juniper Systems), to measure grain weight and moisture content. After adjusting yield to a 14% moisture, grain yield (GY) was converted to t ha^−1^.

The data were analyzed in each environment using the following linear mixed model:

(1) $$y=1\mu +Z_{1}h+Z_{2}r+e$$

where **y** is the vector of phenotypes; **μ** is an intercept; **h** is a random effect of hybrid, $h∼N(0,\sigma_{h}^{2}I)$, **r** is a random effect of replicates, $r∼N(0,\sigma_{r}^{2}I)$; **e** is the vector of residuals, $e∼N(0,\sigma_{e}^{2}I)$; **1** is a vector of ones; **Z_1_** and **Z_2_** are incidence matrixes; , , and are variance components for hybrids, replicates, and residuals, respectively. Model 1 (Equation 1) was extended to incorporate environment and G × E interaction effects for performing a combined analysis. Environments were defined as a year and location combination, hybrid effects were partitioned into GCA and SCA, and G × E effects were represented by the interaction between GCA and SCA with the environment as follows:

(2) $$\begin{array}{ccc} & & y=1\mu +Z_{1}f+Z_{2}m+Z_{3}\mathrm{fm}+Z_{4}s+\;Z_{5}\mathrm{fs}+Z_{6}\mathrm{ms}\\ & & \;+\;Z_{7}\mathrm{fms}+Z_{8}r\left(s\right)+e \end{array}$$

where **f** is a vector of GCA effects of females, $f∼N(0,\sigma_{f}^{2}I)$; **m** is a vector of GCA effects of male, $m∼N(0,\sigma_{m}^{2}I)$; **fm** is a vector of SCA effects of hybrid combinations, $\mathrm{fm}∼N(0,\sigma_{fm}^{2}I)$; **s** is a vector of environmental effects, $s∼N(0,\sigma_{s}^{2}I)$, **fs** is a vector of the interaction effect between GCA of female and environment, $\mathrm{fs}∼N(0,\sigma_{fs}^{2}I)$; **ms** is a vector of the interaction effect between GCA of male and environment, $\mathrm{ms}∼N(0,\sigma_{ms}^{2}I)$, **fms** is a vector of the interaction effect between SCA of hybrid combinations and environment, $\mathrm{fms}∼N(0,\sigma_{fms}^{2}I)$; **r(s)** is the vector of replication effect nested within environment, $r(s)∼N(0,\sigma_{r(s)}^{2}I)$; **Z_1_**, **Z_2_**, **Z_3_**, **Z_4_**, **Z_5_**, **Z_6_**, **Z_7_**, and **Z_8_** are incidence matrixes for effects.

Variance components were estimated via restricted maximum likelihood (REML) method (Patterson & Thompson, 1971), and significance assessed by the likelihood ratio test (LRT) at a 5% significance level using the *ranova* function of the *lmerTest* R package (Kuznetsova et al., 2017). To assess phenotypic data, the *lmer* function of the *lme4* R package was applied (Bates et al., 2015). From the variance component estimates, repeatability (i.e., broad sense heritability) at each environment was calculated as $R=\sigma_{h}^{2}/\left[\sigma_{h}^{2}+\left(\sigma_{e}^{2}/r\right)\right]$, whereas, for combined environments,

$R=\frac{\sigma_{h}^{2*}}{\sigma_{h}^{2*}+\frac{\sigma_{hs}^{2*}}{t}+\frac{\sigma_{e}^{2}}{tr}}$

where *t* is the number of environments, *r* is the number of replications, $\sigma_{h}^{2*}=\sigma_{f}^{2}+\sigma_{m}^{2}+\sigma_{fm}^{2}$and $\sigma_{hs}^{2*}=\sigma_{fs}^{2}+\sigma_{ms}^{2}+\sigma_{fms}^{2}$. To assess the quality of the experiment, coefficient of variation (CV_e_) was calculated as

$CV_{e}=\frac{\sqrt{\sigma_{e}^{2}}}{\bar{x}}$

where $\bar{x}$ is the mean of a given trait. Finally, the best linear unbiased estimates (BLUEs) of hybrids were calculated for each trait using Equation 1 via the solution of Henderson's mixed equations (Henderson, 1984), assuming hybrids as fixed. The BLUEs were later employed to develop genomic prediction models and calculate the Pearson's correlation between traits.

### 2.2 Genotypic data

Genotyping‐by‐sequencing (GBS) and DNA extraction were conducted according to Morishige et al. (2013). In brief, seed from the parental lines were germinated in a greenhouse and DNA extracted from ∼14‐day‐old leaf tissue. Illumina template libraries were prepared by digesting each DNA sample with the restriction enzyme *Ngo*MIV. Fragments from each DNA sample were ligated to unique identifier barcodes; samples were pooled together for PCR‐amplification, and templates sequenced on an Illumina HiSeq 2500 sequencer. Sequences were mapped to the *Sorghum bicolor* BTx623 reference genome (Sbicolor v3.1.1) downloaded from Phytozome (https://genome.jgi.doe.gov/portal/pages/dynamicOrganismDownload.jsf?organism=Phytozome), and single nucleotide polymorphisms (SNPs) detected using the CLC Genomics Workbench v20 (CLC Bio). Genomic polymorphisms scored in at least 25% of the parental lines were called SNPs, and a total of 117,582 SNPs were identified. Markers with more than 5% missing values were removed. For the remaining markers, missing values were imputed using FastPHASE (Scheet & Stephens, 2006), and SNPs with minor allele frequency (MAF) < 5% were removed. After quality control, 35,546 SNP were available for further analysis.

Genomic relationship matrixes for female and male parents were computed using markers. Matrices were computed as follows: 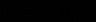, where **W_k_** is a matrix of markers centered and standardized, *r* is the number of markers, k∈{f,m} (Technow et al., 2014; Lopez‐Cruz et al., 2015).

Alternatively, the genetic distance between parents was calculated on a pairwise basis using the Prevosti's absolute genetic distance (Prevosti et al., 1975) implemented in *poppr* R package (v2.8.6; Kamvar et al., 2014). Distances were computed based on the differences in the frequencies of SNPs as

$D=\frac{1}{2r}\sum_{j=1}^{r}\sum_{k=1}^{s(j)}\left|p_{1jk}-p_{2jk}\right|$

where r is the number of SNPs considered, p_1_ *_jk_* is the frequency of the SNP *k* in the chromosome j in the first parent, and p_2_ *_jk_* is the corresponding value in the second parent. A principal coordinate analysis (PCoA) of the genetic distance was plotted to represent the genetic relatedness of parents. Clustering analysis was performed using the function *CLARA* (Struyf et al., 1997) from R packages *ggplot2* (Wickham, 2016) and *ggfortify* (Tang et al., 2016).

### 2.3 Statistical models

Kernel‐based Bayesian GBLUP models (GB) and classical GCA‐SCA–based models under a Bayesian Ridge Regression (BRR) framework were fitted to predict yield, height, and days to anthesis of grain sorghum hybrids. These models differed on the (co)variance matrix related to GCA and SCA effects; genomic models included genomic information to account for heterogeneity within parents and hybrids (i.e., accommodate population structure), whereas classical GCA‐SCA–based models replaced genomic information with the identity matrix (i.e., without the genomic kernel). The GB and BRR models were developed based on genetic main effects of GCA (seed and males) and SCA at three distinct levels of complexity: (a) for a single environment, (b) multi‐environment, and (c) multi‐environment plus the G × E interaction effect.

#### 2.3.1 Single‐environment main genetic effect model (SM)

The linear model adjusted the BLUE of hybrids derived from Model 1 (Equation 1) separately for each environment and included GCA and SCA genetic effects as follows:

(3) $$y\;=1\mu +Z_{1}f+Z_{2}m+Z_{3}h+e$$

where **y** = [**y_1_**, … , **y*_n_***] is the response vector, and **y*_n_*** represents the observation in the *i*th hybrid (*i* = 1,…, *n*) in each environment; **μ** is an intercept, **f** is the vector of GCA effects for females, $f∼N(0,\sigma_{fj}^{2}G_{f})$, where *j* represents environment; **m** is a vector of GCA effects for males, $m∼N(0,\sigma_{mj}^{2}G_{m})$; **h** is a vector of SCA effects for hybrid combinations, $h∼N(0,\sigma_{hj}^{2}H)$; e is a vector of residuals, $e∼N(0,\sigma_{ej}^{2}I)$, **1** is a vector of ones; **Z_1_**, **Z_2_**, and **Z_3_** are incidence matrixes for females, males, and hybrids, respectively; and **G_f_**, **G_m_**, and **H** are the GRM for females, males, and hybrids, respectively. The elements of matrix **H** were calculated in silico by the Kronecker product of **G_f_** and **G_m_** as **H** = **G_f_** ⊗ **G_m_** (Technow et al., 2014; Acosta‐Pech et al., 2017). This model was also used to build prediction models using the grand mean of hybrids across environments (i.e., mean of the BLUEs). For BRR, Model 3 (Equation 3) was fitted without incorporating genomic kernels, thus the GRM of parents and hybrids were replaced by an identity matrix.

#### 2.3.2 Multi‐environment main genetic effect model (MM)

One multi‐environment model that includes the fixed effects of environments and the random genetic effects across environments is presented by Bandeira e Sousa et al. (2017) as follows

(4) $$y=1\mu +Z_{s}\beta +Z_{1}\;f+Z_{2}m+Z_{3}h+e$$

where **y** = [**y_1_**, … **y_j_**, … **y_s_**] is the vector of observations of hybrids (*i* = 1, … , *n_j_*) in the *j*th environment (*j* = 1, … , *s*); **β** is a vector of the environmental effects, $\beta ∼N(0,\sigma_{\beta }^{2}I)$; and **Z_s_** is the incidence matrix that connects hybrids to phenotypes for each environment. Random effects are described in Model 3 (Equation 3) and are assumed to be equal across environments (see Lopez‐Cruz et al., 2015). For BRR, Model 4 (Equation 4) was fitted without incorporating genomic kernels to genetic main effects.

#### 2.3.3 Multi‐environment main genetic effect model plus G × E (MMGE)

The following model (Equation 5) is an extended version of Model 4 (Equation 4) to include G × E effects as proposed by Acosta‐Pech et al. (2017). These authors considered the incorporation of G × E effects (**u**) by applying the Hadamard product between genetic main effects (i.e., **G_f_**, **G_m_**, and **H**) and the environmental effect. The model is given as follows:

(5) $$y=Z_{s}\;\beta +Z_{1}f+Z_{2}m+Z_{3}h+u_{f}+u_{m}+u_{h}+e$$

where **u_f_** is a random vector to include the interaction between GCA of females and environment, $u_{f}∼N\left(0,\sigma_{\mathrm{fs}}^{2}V_{f}\right)$; **u_m_** is a random vector that models the interaction between males and environment, $u_{m}∼N\left(0,\sigma_{\mathrm{ms}}^{2}V_{m}\right)$; **u_h_** is a vector that takes into account the interaction between hybrids and environment, $u_{h}∼N\left(0,\sigma_{\mathrm{hs}}^{2}V_{h}\right)$; **V_f_**, **V_m_**, and **V_H_** are variance–covariance matrices that associate each respective genomic‐main‐effect × environment variance component, i.e., $\sigma_{\mathrm{fs}}^{2}$, $\sigma_{\mathrm{ms}}^{2}$, and $\sigma_{\mathrm{hs}}^{2}$. The variance–covariance matrix was calculated as **V_f_** = **Z_1_G_f_Z_1_** #**Z_s_Z_s_**, **V_m_** = **Z_2_G_m_Z_2_** #**Z_s_Z_s_**, and **V_h_** = **Z_3_HZ_3_** #**Z_s_Z_s_** where *#* stands for the Hadamard product (see Acosta‐Pech et al. (2017) for further details about the derivations). For BRR, Model 5 (Equation 5) was fitted without incorporating genomic kernels to genetic main effects and G × E was calculated as **V_f_ **= **Z_1_Z_s_**, **V_m_** = **Z_2_Z_2_**, and **V_h_** = **Z_1_Z_2_** #**Z_s_Z_s_** for the interaction of GCA_f_, GCA_m_, and SCA with the environment.

### 2.4 Software and cross‐validation scheme

The GB and BRR models were fit using Bayesian methods implemented in the *BGLR* statistical package (Pérez & de los Campos, 2014) available for R (R Core Team, 2020). Inferences were based on 10,000 Gibbs sampler iterations with a *burn‐in* of 5,000, and a *thin* of 5. For BRR models (i.e., classical GCA‐SCA–based models), all effects assumed Gaussian prior distribution and included an identity (**I**) matrix to structure the data set, whereas, for GB models, only environmental effect followed this assumption. The remaining effects assumed a Gaussian prior distribution and applied the kernel‐based method to create the appropriate variance–covariance structures (Pérez & de los Campos, 2014).

To validate models and estimate the predictability of parents, a simple leave‐one‐out cross‐validation (CV) scheme was applied following Basnet et al. (2019). For that, seed parents in hybrid combinations were removed, and models were then trained with the remaining records where that seed parent was absent, i.e., predicting hybrids of unobserved females (T1F). The same method was applied to pollinator parent (T1M). Further, seed and pollinator parents were removed simultaneously to predict hybrids (T0MF). Finally, T1F and T1M were applied to train models considering just T × T and K × K hybrids under subtropical and temperate environments (Supplemental Table S2). Subtropical and temperate environments were assumed to be the target environments for the respective T × T and K × K hybrids because each set of hybrids derived from parents adapted to each respective environment. The prediction accuracy (*r*) was estimated as the Pearson's correlation between observed and predicted values of hybrids.

### 2.5 Coincidence index

The coincidence index (CI) originally proposed by Hamblin and Zimmermann (1986) aimed to assess the proportion of coincident genotypes selected under two different planting systems assuming a specific selection intensity (SI). Herein, a modified version of such CI is proposed to calculate the optimum SI that maximizes the CI between observed and predicted hybrids utilizing a genomic‐enabled prediction model. Calculations of CI was performed using GB‐MMGE and BRR‐MMGE under T0MF. For that, simulated SI values ranging from 1 to 50% were considered and CI computed as:

$$\mathrm{CI}\;=\frac{C-R}{T-R}$$

where *C* is the number of coincident hybrids between observed and predicted values; *T* is the number of observed hybrids selected according to the SI; and *R* is the number of expected hybrids selected by chance, i.e., a fraction of *T* that also varies depending on a SI. For instance, considering a population of 100 hybrids, if SI = 10; then *T* = 10 (100 × 0.10), *R* = 1(10 × 0.10), and *C* will depend on the number of coincident hybrids between observed and predicted values. If *C* = 2, then CI = 1/9 = 11%.

## 3 RESULTS

### 3.1 Phenotypic analysis

**FIGURE 1:**
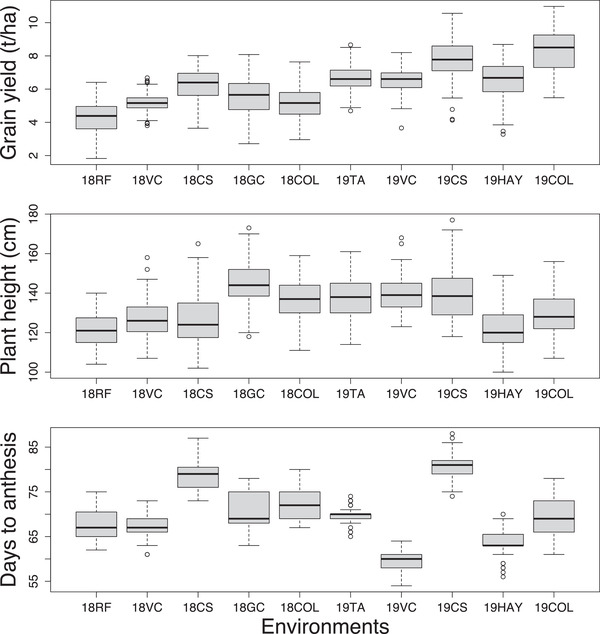
Boxplot of best linear unbiased estimates (BLUEs) for grain yield (t ha^−1^), plant height (cm), and days to anthesis in each environment. Environments are indicated as the combination of year and location where hybrid trials were conducted. Designations for environments are as follows: Monte Alto 2018 (18RF), Victoria 2018 (18VC), College Station 2018 (18CS), Garden City 2018 (18GC), Colby 2018 (18COL), Taft 2019 (19TA), Victoria 2019 (19VC), College Station 2019 (19CS), Hays 2019 (19HAY), and Colby 2019 (19COL)

**FIGURE 2:**
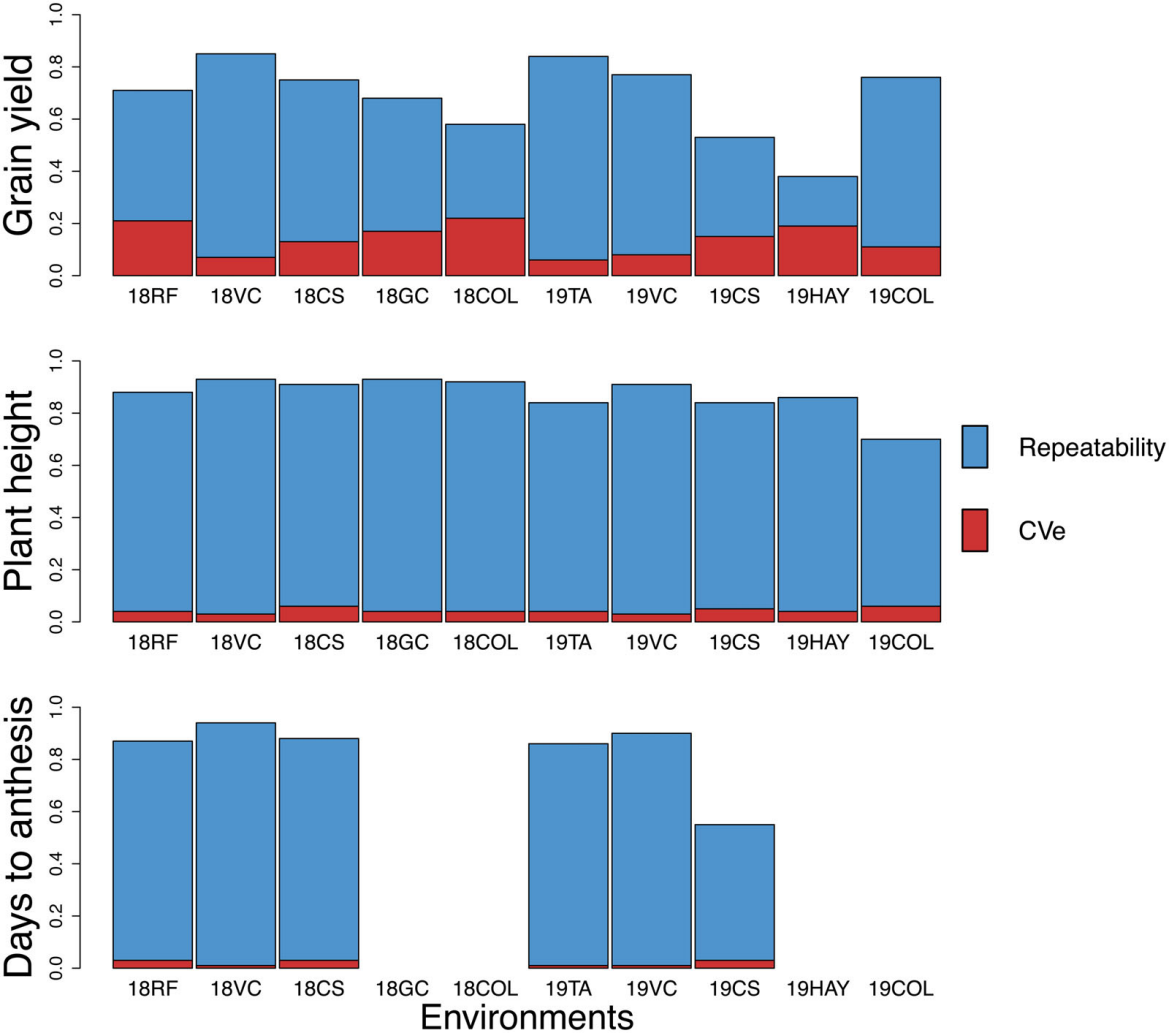
Repeatability and experimental coefficient of variation (CVe) estimates for grain yield, plant height, and days to anthesis in each environment. Environments are indicated as the combination of year and location where hybrid trials were conducted. Designations for environments are as follows: Monte Alto 2018 (18RF), Victoria 2018 (18VC), College Station 2018 (18CS), Garden City 2018 (18GC), Colby 2018 (18COL), Taft 2019 (19TA), Victoria 2019 (19VC), College Station 2019 (19CS), Hays 2019 (19HAY), and Colby 2019 (19COL)

The average of the best linear unbiased estimate (BLUE) of hybrids across environments varied significantly for all traits (Figure 1). Values for hybrid GY ranged from 1.83 to 10.98 t ha^−1^, from 100 to 177 cm for PHT, and from 54 to 88 d for DA. The lowest mean hybrid GY occurred in 2018 Monte Alto, TX, and the highest occurred in 2019 Colby, KS. The repeatability of PHT and DA were consistently high whereas the repeatability of GY was lower and varied more across environments (Figure 2). Coefficient of variation (CVe) indicated that data variation within locations were typical for sorghum grain hybrid yield trials (Figure 2).

The likelihood ratio test indicated that all genetic effects and their interactions with the environment were significant (Table 1). As expected, the environmental effect was highly significant, accounting for the majority of variation for GY (50%) and DA (76%). Although the majority of the genetic variation was associated with GCA, SCA was also significant, indicating the importance of specific hybrid combinations to maximize productivity.

**TABLE 1:** Variance components, repeatability, and experimental coefficient of variation (CVe) estimates derived from the combined analysis for grain yield (GY), plant height (PHT), and days to anthesis (DA). Likelihood ration test (LRT) indicate the significance of effects. The percentage of the total variation (%) for each variance component is shown with its respective estimate

	GY		PHT		DA	
Variance components	Estimate	%	Estimate	%	Estimate	%
(Hybrid)	(0.315)	(10.4)	(89.6)	(42.5)	(6.0)	(11.9)
GCA_f_	0.128***	4.2	34.2***	16.2	2.5***	5.0
GCA_m_	0.150***	4.9	47.1***	22.3	3.2***	6.4
SCA	0.037***	1.2	8.3***	3.9	0.3***	0.5
Environment	1.430**	47.5	67.9***	31.9	37.8***	75.0
(Hybrid × Env)	(0.382)	(12.7)	(18.3)	(8.7)	(2.9)	(5.6)
GCA_f_ × Env	0.175***	5.8	5.2***	2.5	1.3***	2.6
GCA_m_ × Env	0.157***	5.2	9.3***	4.4	1.3***	2.5
SCA × Env	0.050*	1.6	3.7***	1.7	0.2*	0.4
Rep (Env)	0.082***	2.7	3.3***	1.6	0.6***	1.1
Residual	0.796	26.5	32.5	15.4	3.2	6.3
Repeatability	0.819		0.966		0.934	
CVe	0.144		0.043		0.024	

* Significant at the 0.05 probability level.

** Significant at the 0.01 probability level.

*** Significant at the 0.001 probability level.

Pearson's correlations between traits were assessed for each environment and across environments assuming a 95% confidence interval. Correlations between traits were somewhat inconsistent across individual environments, but correlations across all environments were significant and positive between GY and PHT (0.17 ± 0.06), and PHT and DA (0.15 ± 0.06). Overall correlations across all environments indicate that taller hybrid grain sorghums are generally higher in yield; however, this trend is mitigated by environmental stresses in selected environments (Rooney et al., 2003).

### 3.2 Genetic distance between inbred parents

**FIGURE 3:**
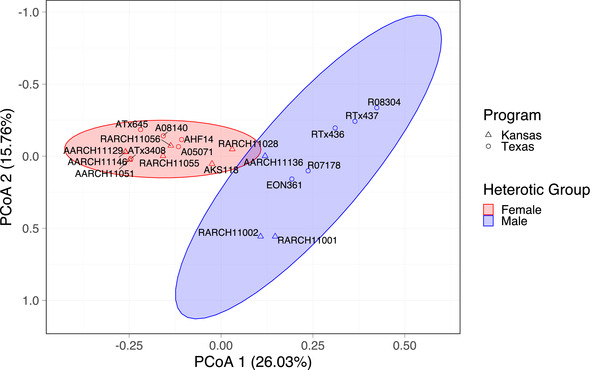
Bidimensional representation of the genetic distance of Texas and Kansas inbred parents used in the factorial mating scheme. Principal Coordinate Analysis (PCoA) display the first and the second axis with its correspondent % of genetic variance explained by each principal coordinate. Clusters represent each heterotic group base on SNP markers

The plotted Principal Coordinates (PCoA) explained almost 42% of the total genetic variation among inbred parents in this study (Figure 3). For the most part, the A‐line parents were a distinct group although some R‐lines clustered within this group. The R‐lines within the A‐line cluster originated from the Kansas breeding program, and their pedigrees reveal the presence of B‐line lineage. Similarly, one A‐line from Kansas clustered within the male group, indicating some R‐line lineage within its pedigree. Overall there was greater genetic variation in the R‐lines, which has been well documented in previous studies (Menz et al., 2004; Crozier et al., 2020). In general, the genetic distance within Texas R‐lines was greater than within Kansas R‐lines (Figure 3).

### 3.3 Predictive ability across parents, traits, and models to predict hybrids

The predictive ability of parents to predict hybrids varied substantially across traits and models. Across their hybrids, KS118 and R07178 had the highest predictabilities for an A‐line and R‐line, respectively. As a group, the A‐lines had higher prediction ability than the R‐lines. Concerning traits, predictions for PHT and DA were better than for GY, regardless of the genetic group (Table 2).

**TABLE 2:** Prediction accuracy of classical GCA‐SCA–based Bayesian Ridge Regression (BRR) and kernel‐based Bayesian GBLUP (GB) models for single environment (SM), multi‐environment (MM), and multi‐environment plus G × E effect (MMGE) from leave‐one‐female‐out, and leave‐one‐male‐out cross‐validation schemes for grain yield, plant height, and days to anthesis. The average prediction ability across all A‐ and R‐lines represents the overall model predictability. Higher predictabilities within BRR and GB approaches are in black boldface, whereas the best predictability between approaches are in blue boldface

		BRR			GB		
Parents		SM^*^	MM	MMGE	SM^*^	MM	MMGE
**Grain yield**							
A‐line	A05071	**.913**	.496	.492	**.923**	.496	.652
	A08140	**.792**	.455	.452	**.785**	.466	.588
	AARCH11051	**.851**	.666	.666	**.696**	.668	.673
	AARCH11129	**.616**	.300	.302	**.648**	.306	.373
	AARCH11136	**.633**	.455	.458	.597	.463	**.689**
	AARCH11146	.446	**.489**	.487	.508	.484	**.625**
	AHF14	.048	**.468**	.467	.040	.457	**.597**
	AKS118	**.732**	.626	.619	**.748**	.627	.706
	ATx3408	.434	**.528**	.521	.388	.526	**.681**
	ATx645	.348	.490	**.494**	.396	.500	**.667**
Mean		.581	.497	.496	.573	.499	**.625**
R‐line	EON361	.208	.445	**.518**	.251	.443	**.546**
	R07178	**.881**	.487	.615	**.904**	.490	.640
	R08304	.370	.335	**.481**	.459	.347	**.508**
	RARCH11001	.136	.413	**.550**	.463	.422	**.624**
	RARCH11002	.231	.422	**.664**	.481	.438	**.712**
	RARCH11028	**.548**	.251	.467	**.559**	.260	.473
	RARCH11055	.503	.556	**.623**	.318	.554	**.577**
	RARCH11056	**.597**	.381	.568	.282	.387	**.563**
	RTx436	**.583**	.415	.497	**.623**	.407	.488
	RTx437	**.640**	.447	.614	**.636**	.447	.575
Mean		.470	.415	.560	.498	.420	**.571**

**Plant height**	
A‐line	A05071	.730	**.789**	**.789**	.752	.800	**.845**
	A08140	.719	**.773**	.772	.817	.806	**.851**
	AARCH11051	**.874**	.853	.854	**.918**	.866	.912
	AARCH11129	**.817**	.805	.802	.846	.820	**.872**
	AARCH11136	**.910**	.779	.777	**.882**	.774	.816
	AARCH11146	**.921**	.744	.745	**.921**	.733	.791
	AHF14	**.745**	.732	.733	.781	.750	**.803**
	AKS118	**.909**	.739	.740	**.926**	.748	.816
	ATx3408	.829	**.839**	.835	.817	.841	**.898**
	ATx645	.620	.719	**.720**	.694	.752	**.825**
Mean		.807	.777	.777	.835	.789	**.843**
R‐line	EON361	.397	.607	**.614**	.525	.651	**.676**
	R07178	.564	.765	**.781**	.764	.809	**.841**
	R08304	.710	.805	**.824**	.755	**.825**	**.825**
	RARCH11001	.759	.714	**.771**	.715	.764	**.830**
	RARCH11002	**.827**	.783	.821	.836	.809	**.858**
	RARCH11028	.637	.640	**.683**	.632	.651	**.693**
	RARCH11055	.697	.829	**.831**	.685	.802	**.809**
	RARCH11056	**.765**	.722	.757	**.805**	.735	.774
	RTx436	.672	.690	**.728**	.661	.699	**.738**
	RTx437	.656	.768	**.798**	.697	.776	**.805**
Mean		.668	.732	.761	.707	.752	**.785**

**Days to anthesis**							
A‐line	A05071	.694	**.720**	**.720**	.660	.714	**.764**
	A08140	.489	**.707**	.705	.404	**.701**	.694
	AARCH11051	.622	**.644**	**.644**	.600	.656	**.760**
	AARCH11129	**.821**	.589	.584	**.835**	.596	.685
	AARCH11136	**.867**	.779	.776	**.865**	.777	.863
	AARCH11146	**.837**	.741	.739	.781	.739	**.808**
	AHF14	.794	.802	**.806**	.806	.809	**.831**
	AKS118	**.834**	.776	.777	**.864**	.779	.839
	ATx3408	.663	**.811**	**.811**	.704	.811	**.849**
	ATx645	.654	**.555**	**.555**	.562	.543	**.617**
Mean		.728	.712	.712	.708	.712	**.771**
R‐line	EON361	**.760**	.651	.730	**.795**	.646	.738
	R07178	.798	.813	**.833**	.824	.813	**.833**
	R08304	.750	.769	**.779**	.781	.770	**.799**
	RARCH11001	.490	.679	**.747**	.539	.683	**.753**
	RARCH11002	.496	.690	**.716**	.542	.696	**.745**
	RARCH11028	.699	.611	**.739**	.662	.607	**.739**
	RARCH11055	.201	.459	**.551**	.209	.489	**.630**
	RARCH11056	**.919**	.677	.754	**.933**	.708	.830
	RTx436	**.888**	.616	.657	**.877**	.609	.673
	RTx437	**.939**	.725	.719	**.950**	.727	.734
Mean		.694	.669	.723	.711	.675	**.747**

^a^ Predictions for the environment 19CS.

The inclusion of the environmental effect (MM) reduced the accuracy of models to predict hybrids in most cases whereas the inclusion of the G × E effect (MMGE) considerably increased the prediction ability of models. These increases in prediction ability of models were greatest when parental genomic information was included (GB). For GB, the superiority of models that included the G × E effects was consistent across traits (Table 2).

As expected, the best predictability of hybrid performance occurred when the grand mean of BLUEs was used to train models (data not shown). This likely happened because the number of observations available to predict the grand average of hybrids increased 10‐fold compared with predicting hybrid performance for each location. Although training models with the grand mean of BLUEs effectively improved predictability, this strategy is problematic when significant G × E effects exist (as in this case) due to changes in the rank of hybrids across environments.

**FIGURE 4:**
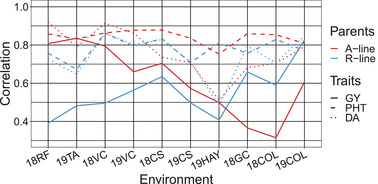
Genomic‐enabled prediction ability of kernel‐based Bayesian GBLUP (GB) model from the average prediction ability of A‐ and R‐lines in each environment for grain yield (GY), plant height (PHT), and days to anthesis (DA). Designations for environments are as follows: Monte Alto 2018 (18RF), Victoria 2018 (18VC), College Station 2018 (18CS), Garden City 2018 (18GC), Colby 2018 (18COL), Taft 2019 (19TA), Victoria 2019 (19VC), College Station 2019 (19CS), Hays 2019 (19HAY), and Colby 2019 (19COL)

The average predictability of parents for all hybrid traits was assessed within each environment using the SM model (Figure 4). The A‐ and R‐lines had similar and generally high predictabilities for PHT and DA across environments (Figure 4). For hybrid GY, predictability varied; A‐lines had higher predictability in subtropical environments, whereas the R‐lines predicted hybrids more accurately in temperate environments. The exception occurred in 19HAY, where females presented higher prediction accuracy than males.

### 3.4 Predictive ability across parents and target environment using GB‐MMGE

The hybrids derived from Texas parents (T × T) were better predicted when genomic prediction models were developed with data from subtropical environments except for PHT in the T1M cross‐validation scheme (when males are removed). Similar results occurred when genomic prediction models were developed using phenotypic records derived from temperate environments to predict K × K hybrids. In this case, the accuracy was higher for all traits and cross‐validation schemes (Table 3). These observations likely reflect the adaptability and stability of inbred lines bred in the respective target environments. Hybrids derived from lines bred for the target environment benefit from positive covariances existing between the loci and the target environment, which thereby increases the accuracy of genomic prediction models.

**TABLE 3:** Genomic‐enabled prediction accuracies under kernel‐based Bayesian GBLUP models (GB) approach for parents at subtropical and temperate environments using the multi‐environment plus G × E effect model (MMGE) for grain yield (GY), plant height (PHT), and days to anthesis (DA). Cross‐validation scheme (T1F and T1M) for each set of hybrids (T × T and K × K) were applied for each trait within each subtropical and temperate environment. Higher predictabilities are depicted in boldface

Hybrids	Parents	Subtropical environment			Temperate environment		
		GY	PHT	DA	GY	PHT	DA
T × T (T1F)	A05071	.720	.837	.852	.442	.610	.625
	A08140	.387	.822	.870	.489	.151	.497
	AHF14	.589	.842	.887	.276	.649	.814
	ATx3408	.470	.719	.831	.463	.832	.913
	ATx645	.532	.735	.831	.745	.214	.533
	Mean	**.540**	**.791**	**.854**	.483	.491	.677
T × T (T1M)	EON361	.536	.657	.859	.532	.688	.742
	R07178	.451	.830	.866	.165	.884	.829
	R08304	.066	.798	.846	.427	.836	.689
	RTx436	.224	.899	.795	‐.076	.929	.598
	RTx437	.294	.854	.883	.374	.834	.735
	Mean	**.314**	.807	**.850**	.285	**.834**	.718
K × K (T1F)	AARCH11051	.388	.778	.699	.451	.945	.815
	AARCH11129	.420	.847	.765	.121	.840	.704
	AARCH11136	.203	.901	.913	.758	.933	.933
	AARCH11146	.326	.854	.792	.363	.913	.838
	AKS118	.521	.698	.804	.394	.823	.950
	Mean	.372	.816	.795	**.417**	**.891**	**.848**
K × K (T1M)	RARCH11001	.588	.742	.914	.779	.938	.929
	RARCH11002	.453	.887	.796	.776	.940	.867
	RARCH11028	.432	.577	.630	.297	.814	.824
	RARCH11055	.241	.886	.208	.465	.946	.431
	RARCH11056	.196	.836	.549	.242	.883	.635
	Mean	.382	.786	.619	**.512**	**.904**	**.737**

### 3.5 Impact of genomic and classical GCA‐SCA–based models on the coincidence index

**FIGURE 5:**
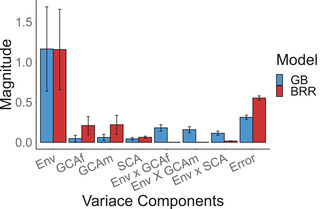
Variance component estimates and standard deviations for grain yield using best linear unbiased estimate (BLUE) under multi‐environment genomic (GB) and classical GCA‐SCA–based (BRR) models. The graph includes estimates for environmental effect (Env), general combining ability of females (GCAf), general combining ability of males (GCAm), specific combining ability (SCA), interaction effect between environment and combining abilities (Env × GCAf, Env × GCAm, Env × SCA), and error term

**FIGURE 6:**
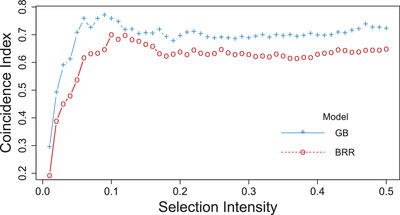
Coincidence index and selection intensity for grain yield using multi‐environment genomic (GB) and classical GCA‐SCA–based (BRR) models under cross‐validation scheme T0MF (both male and female is taken out simultaneously)

Means across environments were used to calculate variance components for hybrid grain yield to compare genomic (GB) and classical GCA‐SCA–based (BRR) models (Figure 5). In both models, the majority of the variation is attributed to the environment, but the partition of the variation differed between models. For example, GCA for females and males was lower in GB; alternatively, BRR did not capture G × E (e.g., Env × GCA). Both models showed similar estimates for SCA but the Env × SCA estimate was considerably smaller in BRR than GB. The error component in the GB model was lower than the same in BRR. The generally lower standard deviations in GB are likely the reason for the higher precision of the variance component estimates. Finally, the deviance information criteria (DIC) for BRR was higher than for GB indicating that multi‐environment models to predict hybrid performance should include the GRM. The robustness of GB‐MMGE increased the CI at any SI so that GB‐MMGE was efficient even when SI assumed values <10% (Figure 6). The highest CI values (∼79%) were obtained when SI assumed values around 9%. After a slight decay in the CI with an increase in SI, CI maintained a value of ∼70% for the GB‐MMGE model, and ∼60% for the BRR‐MMGE model.

## 4 DISCUSSION

### 4.1 Predicting grain sorghum hybrid performance

Bernardo (1994) first described the concept of predicting hybrids with the application of GCA and SCA genetic effects using the Best Linear Unbiased Prediction (BLUP) model in maize. Technow et al. (2012) developed a simulation study based on a maize breeding program having complementary heterotic groups to assess genomic prediction of hybrid performance using different models; results supported the application of models based on GCA and SCA to suitably include genetic effects. In the present study, we extend that concept of predicting hybrids using GCA and SCA to a hybrid cereal crop that lacks the extensive resources of maize, and we expanded the prediction models to include genomic information and G × E interaction effects under a kernel‐based hierarchical Bayesian framework. Further, the results demonstrate the importance of including GCA and SCA effects to effectively account for the inherent genetic structure that exists in all hybrid breeding populations, including but not limited to sorghum. Both classical GCA‐SCA–based and genomic methods successfully predicted grain sorghum hybrid performance and the application of kernel‐based Bayesian GBLUP models (GB) increased the prediction ability of the models. Although the inclusion of the G × E interaction effects in GB models led to consistently higher prediction accuracies, the same trend was not observed in BRR models.

### 4.2 Advantages of using kernel‐based models

The present implementation of kernel‐based regression models featured practical advantages relative to the use of standard genomic models in the computational process (Crossa et al., 2011), which included a significant reduction with issues related to data dimensionality (e.g., when the number of markers considerably exceeds the number of genotypes) (Gianola & de los Campos, 2008). Additionally, kernel‐based regression models can be used with nearly every information set (e.g., covariates, strings, images, and graphs) (de los Campos et al., 2010; Crossa et al., 2011). For instance, parametric and semi‐parametric kernels accommodate the genomic relationship matrix to represent identical‐by‐state similarities among parents (de los Campos et al., 2009). This creates a unique opportunity for breeders to account for genetic relatedness among genotypes without tracing genealogy (Morota & Gianola, 2014). Nonetheless, if molecular marker datasets are not available, pedigree information can also be used as a kernel. Models based on marker information manifested higher predictive ability than pedigree‐based models in studies involving wheat and maize data (Crossa et al., 2010). Such results exist because pedigree can only explain the expected degree of genetic similarity, but it cannot consider segregation distortion. In addition, dense molecular marker datasets can account for the realized degree of genetic similarity, i.e., the mendelian sample. The linear kernel applied herein that included the genetic relationship among divergent elite grain sorghum parents was effective in adjusting the degree of genetic similarity.

The application of the genomic relationship matrix (VanRaden, 2008) via kernel‐based framework is also critical in hybrid prediction models because of its subtle ability to include population structure in genomic‐enabled prediction models. de los Campos et al. (2015) demonstrated that the inclusion of multi‐bred or sub‐populations is adequate to increase the size of training sets when heterogeneity is modeled. Technow et al. (2013) increased the prediction accuracy of models when assessing northern leaf blight resistance in maize by combining data from heterotic groups. The application of kernels appears to be especially relevant for predicting hybrids using GCA and SCA due to the chance of suitably incorporating population structure to each main genetic effect. Herein, kernels representing the genetic (co)variance between inbred parents were applied to each specific genetic effect. Genetic similarities within A‐ and R‐lines were modeled by unique kernels to adjust the GCA of females and males, respectively. Additionally, the SCA effect was generated in silico by the Kronecker product between the A‐ and R‐lines relationship matrix (de los Campos et al., 2015). This process adequately accounted for heterogeneity and genetic main effect simultaneously, which increased the prediction accuracy of the models. It is important to note that the predictability greatly benefited from the inclusion of kernels especially when A‐lines were involved in the cross‐validation scheme (T1F). This is likely due to the higher degree of similarity among A‐lines compared to among R‐lines. The limited genetic variability present in the A‐lines relative to the R‐lines reflects the longer timeline and difficulty associated with developing high‐performing A‐lines. Thus, kernels make it possible to “borrow” information from closely related parents resulting in the higher predictabilities reported herein.

Another advantage of the kernel‐based genomic prediction models is the opportunity to include G × E effects using markers. Burgueño et al. (2012) introduced a multi‐environment version of the standard GBLUP model to account for the genetic correlation between wheat lines and environments, and the authors reported a significant increase in prediction accuracy. The application of reaction norm models within the context of genomic predictions exhibited similar results (Jarquín et al., 2014a). When reaction norm models are applied, kernels usually include G × E effects by the interaction between markers and environmental covariables. Acosta‐Pech et al. (2017) applied the reaction norm framework using linear kernels to model GCA, SCA, and their interaction with environments; and their results demonstrated the importance of including each of these effects in the genomic prediction of hybrids. Basnet et al. (2019) extended a similar model to include environmental covariables to describe sites and results support its practice. Costa‐Neto et al. (2020) presented nonlinear kernels to incorporate G × E and nonadditive effects in genomic‐based prediction in multi‐environment trials and suggested its application to predict maize hybrids. In the present study, a linear kernel was applied to model G × E effects using the method presented by Acosta‐Pech et al. (2017), and higher prediction accuracy was observed. It is important to highlight that the inclusion of G × E effects in genomic prediction models increases prediction accuracy because it can borrow information existing not only among the females and males involved in hybrid combinations but can also utilize the genetic correlations among environments.

### 4.3 Factors affecting the prediction accuracy in grain sorghum hybrids

In our cross‐validation studies, we were able to ascertain additional factors that impact the prediction accuracy of models for hybrid performance traits. Results herein demonstrated that prediction accuracy (often defined as the Pearson correlation between the predicted and observed values) of grain sorghum hybrids is affected by repeatability and genetic architecture of the trait, the degree of genetic similarity among parents, the structure of the training set, the method used to perform predictions (genomic or classical GCA‐SCA–based models), and the complexity of models (single or multi‐environments). Of these factors, the repeatability of trait measurement was most critical. For instance, the 19HAY environment had lower prediction accuracies for GY and DA; 19HAY also had the lowest repeatabilities for these traits of any environment. Alternatively, in the same environment, PHT had high repeatability and good prediction accuracy. Similar results have been documented in wheat and maize (He et al., 2016; Alves et al., 2019).

Previous studies have also shown the importance of the genetic architecture in genomic‐enabled predictions (Hayes et al., 2010; Jia & Jannink, 2012; Daetwyler et al., 2013; Fernandes et al., 2018). Traits such as PHT and DA are controlled by few loci compared with GY (Quinby, 1974; Hilley et al., 2017; Casto et al., 2019). Thus, as expected, the prediction accuracy of these traits was higher than for GY. An additional factor influencing prediction accuracy is the precision of measuring PHT and DA compared with GY (Velazco et al., 2019). Because GY is a highly complex quantitative trait, efforts to increase the precision and repeatability of GY estimates are essential and should be implemented.

As discussed previously, higher prediction ability for the T1F cross‐validation scheme (i.e., when females are removed) occurred due to similarity within the female heterotic group. An interesting finding in this study was the positive relation between prediction ability and the structure of the training set; this occurred when the training set involved hybrids tested in environments where the inbred parents were well adapted. Such outcomes are probably due to a natural covariance between the genotypes and their adaptive environmental gradient. The inclusion of environmental covariables to classify sites might capture this covariance and explain such relations (Jarquín et al., 2014a; Costa‐Neto et al., 2020). Additionally, classic GCA‐SCA–based (BRR) and less complex models (single/multiple environments without G × E) had poorer prediction ability compared to GB‐MMGE models due to the aforementioned advantages of kernel‐based models. Several studies have reviewed factors influencing the prediction ability of genomic selection in hybrids (Crossa et al., 2014; Zhao et al., 2015; Wang et al., 2018). Among these factors, the size of the training set and its relationship with the validation population is pivotal because genetic relatedness and variation in the elite germplasm is essential. Therefore, a large reference population that accounts for the genetic variation in the breeding program should produce the most accurate genomic prediction models. Finally, high marker density is also important to ensure that most of the genetic variation is being captured (Daetwyler et al., 2013). Although these effects were not directly assessed herein, they should be considered before developing a training set and initiating genomic selection.

### 4.4 Pre‐screening hybrids for field evaluation

Genomic selection offers the advantage of predicting hybrid performance of new inbreds without field evaluation; this approach allows for a much larger initial screening of new inbred parents without the costly investment in hybrid seed production and phenotypic evaluation. This combination should improve both the efficiency of crop improvement and the rates of genetic gain. Alves et al. (2019) reported on the correlation between pre‐screening intensity and the proportion of the 5% best maize hybrids for different traits and environments. According to their study, the selection of the top 30% of the predicted hybrids was able to identify approximately 85% of the best evaluated hybrids. Herein with sorghum, predictions using the GB‐MMGE model under cross‐validation scheme T0MF (both male and female are removed), showed a coincidence index (CI) of almost 80% when less than the top 10% was selected. These results indicate that a significantly smaller subset of all possible hybrid combinations can be pre‐screened for subsequent field trials. Thus, breeders would evaluate only the promising hybrids, significantly increasing the efficiency of resource allocation.

A typical application of the CI is to determine the efficiency of models to perform indirect selection, assuming a fixed selection intensity. Fernandes et al. (2018) applied the CI to assess the efficiency of multi‐trait, indirect, and trait‐assisted genomic selection for improvement of biomass sorghum using a diverse panel. dos Santos et al. (2020) used CI to assess the merit of early selection in biomass sorghum and proposed a two‐level selection framework to enhance genetic gain per unit of time. Such an approach has proven to be capable of assessing the superiority of models under evaluation. Alternatively, results presented herein demonstrated that the CI can identify the optimum selection intensity for a breeding program.

### 4.5 Implementing genomic prediction and selection in sorghum breeding programs

Since the development of sorghum hybrids, sorghum breeding programs have focused on improving the performance of A‐ and R‐lines in a concurrent and complementary mean with the expectation that hybrid combinations between elite inbred lines derived from complementary heterotic groups will maximize heterosis. There is some evidence that this has occurred over the past 60 yr (Assefa & Staggenborg, 2010; Gizzi & Gambin, 2016; Pfeiffer et al., 2019).

This strategy has increased the performance of hybrids, but these increments have been modest, and continual modification to the production environments has exacerbated the selection priorities in sorghum breeding programs (Monk et al., 2014; Pfeiffer et al., 2019). Technow et al. (2014) proposed a paradigm shift on plant breeding program pipelines designed to develop hybrids using genomic selection to scan the early generation of hybrids and only evaluate promising experimental hybrids at the final stages. In that approach, genomic prediction of hybrid performance allows the program to screen for a larger number of hybrids.

To implement a genomic‐enabled prediction model for a hybrid sorghum breeding program, some changes are needed. For instance, programs would need to increase the number of experimental inbreds and the structure of testcrossing to build training models; this testcrossing will be different than the traditional approaches as described by Rooney (2004). The testcross mating scheme would focus more on hybrids per se rather than estimating GCA. Fristche‐Neto et al. (2018) reported that testcross was the least effective mating design to develop training sets to predict maize‐single crosses and recommended using either a full diallel or a factorial, due to the number of hybrid combinations these methods can develop. Herein, a factorial mating design generated sorghum hybrids and the results suggest that such a mating scheme could become a benchmark for producing experimental hybrids within the context of genomic prediction of sorghum hybrid performance.

Gaynor et al. (2017) proposed a two‐part strategy for the effective use of genomic selection to develop inbred lines in a simulation study; part one focused on identifying new inbreds, and a part two focused on identifying parents for subsequent breeding cycles. In concept, it should be possible to accomplish both objectives simultaneously. Genomic selection can be effective and efficiently implemented in sorghum breeding programs by (a) selecting promising hybrids that will participate in future hybrid trials using the models presented previously, while (b) breeding crosses would be designed by combining the information from the genetic relationship derived from the genotyping data plotted in a PCoA, and the GCA of the tested lines (when applicable), or GEBV (using genomic prediction models to estimate breeding values).

### 4.6 Limitations and prospects

The results in this study were based on a full factorial of 20 inbred parents that produced 100 hybrids that were evaluated in 10 environments. Further research, including more parents with an incomplete set of hybrid combinations, is needed to confirm the utility of classical GCA‐SCA–based and genomic models. Additionally, this study was based on a simple leave‐one‐out cross‐validation scheme, which usually results in higher prediction accuracy due to the relatively larger population size left for the training set. Future studies should consider other cross‐validation schemes that remove higher percentages of parents and hybrids, and contemplate common challenges faced by plant breeders. Moreover, only linear kernels were used to compute the GRM; the assessment of non‐linear kernels must be evaluated for their potential to increase prediction accuracies.

Recently, kernel‐based regression models have included high‐throughput remote‐sensing and phenomics data to perform prediction of hybrids (Cuevas et al., 2019; Lane et al., 2020). Studies on grain sorghum hybrid predictions should incorporate such techniques and evaluate their potential benefits. Moreover, multi‐trait and reaction norm models using environmental covariables are likely to increase the prediction ability of untested grain sorghum hybrids in untested environments; thus, future research should also include these effects.

## 5 CONCLUSION

Classical and genomic hierarchical Bayesian models based on GCA, SCA, and their interaction with the environment can effectively predict relevant agronomic traits in untested grain sorghum hybrids; molecular markers can further improve the efficiency of the breeding program pipeline. The inclusion of genomic information in kernel‐based GBLUP models (GB) suitably incorporated the natural population structure existent in a hybrid crop breeding scheme and, simultaneously, allocated the genetic similarity to each specific genetic main effect (i.e., general and specific combining abilities). The incorporation of the G × E interaction effect in GB methods allowed models to utilize information existing among the females and males involved in hybrid combinations and to exploit the genetic correlations among environments. The application of the G × E interaction effect also permits breeders to develop strategies to deploy specific hybrids to the targeted environment. These procedures are likely to become the standard method to implement genomic selection in sorghum breeding programs for the reasons presented herein. Finally, the suitability of the GB‐MMGE model improves the prediction capacity of grain sorghum hybrids, which permits increasing selection intensity and, ultimately, increasing the rates of genetic gain.

## AUTHOR CONTRIBUTIONS

Jales Fonseca: Conceptualization; Data curation; Formal analysis; Funding acquisition; Investigation; Methodology; Visualization; Writing‐original draft; Writing‐review & editing. Patricia E Klein: Investigation; Writing‐review & editing. Jose Crossa: Data curation; Formal analysis; Methodology; Writing‐review & editing. Angela Pacheco: Data curation; Formal analysis; Writing‐review & editing. Paulino Perez‐Rodriguez: Data curation; Formal analysis; Writing‐review & editing. Perumal Ramasamy: Conceptualization; Investigation; Resources; Writing‐review & editing. Robert Klein: Investigation; Writing‐review & editing. William L Rooney: Conceptualization; Funding acquisition; Investigation; Methodology; Project administration; Resources; Supervision; Writing‐review & editing.

## CONFLICT OF INTEREST

The authors declare that there is no conflict of interest.

## Supporting information

Supplemental Table S1. List of A‐lines and B‐lines from Texas A&M and Kansas State breeding programs used in the factorial mating scheme to develop hybrids.
Supplemental Table S2. Classification of the environments at which hybrid trials were assessed.
