# Supplementary material for: Assessing combining abilities, genomic data, and genotype × environment interactions to predict hybrid grain sorghum performance
Source: Plant Genome. 2021 Aug 9;14(3):e20127. doi: 10.1002/tpg2.20127 (PMC12806885; doi:10.1002/tpg2.20127)
Supplement: Supplementary file 1 [file TPG2-14-e20127-s001.docx]

## Supplemental file

**Supplemental Table S1.** List of A-lines and B-lines from Texas A&M and Kansas State breeding programs used in the factorial mating scheme to develop hybrids. The information includes pedigree, seed color, plant color, and publications describing the material, if applicable.

| **Parents** | **Pedigree** | **Seed** | **Plant** | **Reference** |
| --- | --- | --- | --- | --- |
| **KSU A-lines** |  |  |  |  |
| ARCH11051A&B | ms3tan/ms3/3/TX623B/PI 550610B//01714B | White | Tan | Experimental line |
| ARCH11129BA&B | KP8BS2#16/OPB/3/Tx623 B/PI550610B//01714B | White | Tan | Experimental line |
| ARCH11136A&B | RRSWht-tanB0641701-167B | White | Tan | Experimental line |
| ARCH11146A&B | ms3tan/ms3/3/Tx623B/PI 550610B//01714B | White | Tan | Experimental line |
| KS118A&B | ms3tan//RTx436/PI550610 | White | Tan | Kofoid & Harvey (2005) |
| **KSU R-lines** |  |  |  |  |
| ARCH11001R | 87BH8606-6//ms3/PI 550610/3/SC 414-12ER/PI 550610/4/N310B | White | Tan | Perumal et al. (2019) |
| ARCH11002R | 87BH8606-6//ms3/PI 550610/3/SC 414-12E R/PI 550610/4/N310B | White | Tan | Perumal et al. (2019) |
| ARCH11028R | 2006ISO11(Wht-tan RRS)R | White | Tan | Perumal et al. (2019) |
| ARCH11055R | 9614B/5/ms3tan/BHF14-(B1*Bvar)B/4/ms3//ms3/PI 550610 | Red | Tan | Perumal et al. (2019) |
| ARCH11056R | 9614B/5/ms3 tan/BHF14-(B1*Bvar)B/4/ms3//ms3/PI 550610 | Red | Tan | Perumal et al. (2019) |
| **Texas A&M A-lines** |  |  |  |  |
| A05071 | BDLO357/BHF88 | White | Pigmented |  |
| ATx645 | BTx3042//BTx625/BTx642 | Red | Pigmented |  |
| ATx3408 | BTx631/08PR047 | White | Tan | Mbulwe et al. (2015) |
| AHF14 | BTx643/BTx635 | White | Tan |  |
| A08140 | BTx642//B9411/BTx645 | Yellow | Tan |  |
| **Texas A&M R-lines** |  |  |  |  |
| R08304 | Tx430/00CS765 | Red | Pigmented |  |
| RTx437 | 77CS4/RTx430 | White | Tan | Rooney et al. (2003) |
| R07178 | 91BE7414/R01125 | Red | Tan |  |
| RTx436 | SC120-6-sel/2//Tx7000 | White | Tan | Miller et al. (1992) |
| EON361 | R5646/SC326-6 | White | Tan |  |

**References**

Kofoid, K. D., & Harvey, T. L. (2005). Registration of greenbug resistant sorghum germplasm lines KS 116 A/B through KS 120 A/B. *Crop Science, 45*, 802. <https://doi.org/10.2135/cropsci2005.0802>

Mbulwe, L., Peterson, G. C., Scott-Armstrong, J., & Rooney, W. L. (2015). Registration of sorghum germplasm Tx3408 and Tx3409 with tolerance to sugarcane aphid [*Melanaphis sacchari* (Zehntner)]. *Journal of Plant Registrations, 10*, 51–56. <https://doi.org/10.3198/jpr2015.04.0025crg>

Miller, F. R., Dusek, T. F., Prihoda, K. L., & Rooney, L. W. (1992). Registration of RTx436 sorghum parental line. *Crop Science, 32*, 1518–1518. <https://doi.org/10.2135/cropsci1992.0011183X003200060059x>

Perumal, R., Tesso, T., Kofoid, K. D., Aiken, R. M., Prasad, P. V. V., Bean, S. R., Wilson, J. D., Herald, T. J., & Little, C. R. (2019). Registration of six grain sorghum pollinator (R) lines. *Journal of Plant Registrations, 13*, 113–117. <https://doi.org/10.3198/jpr2017.12.0087crp>

Rooney, W. L., Miller, F. R., & Rooney, L. W. (2003). Registration of RTx437 sorghum parental line. *Crop Science, 43*, 445. <https://doi.org/10.2135/cropsci2003.4450>

**Supplemental Table S2.** Classification of the environments at which hybrid trials were assessed. The information includes production season, average temperature, average precipitation, and adaptation zone. Temperature and rainfall describe averages at the beginning and end of the production season.

| **Location** | **Production season** | **Temperature (°C)** | **Precipitation (mm)** | **Adaptation zone** | **GPS**  **coordinates** |
| --- | --- | --- | --- | --- | --- |
| Monte Alto | Feb - Jun | 18 - 28 | 20 - 58 | Subtropical | 26°21'06.1"N 97°53'50.3"W |
| Taft | Mar - Jul | 16 - 27 | 46 - 64 | Subtropical | 28°00'05.4"N 97°15'12.4"W |
| Victoria | Mar - Jul | 18 - 29 | 56 - 64 | Subtropical | 28°47'24.4"N 96°50'22.6"W |
| College Station | Mar - Jul | 16 - 29 | 66 - 52 | Subtropical | 30°32'56.6"N 96°26'11.5"W |
| Garden City | Jun - Oct | 24 - 14 | 66 - 61 | Temperate | 37°59'21.4"N 100°48'52.5"W |
| Colby | Jun - Oct | 22 - 12 | 61 - 22 | Temperate | 39°22'56.6"N 101°04'45.0"W |
| Hays | Jun - Oct | 24 - 19 | 79 - 32 | Temperate | 38°51'10.8"N 99°20'24.6"W |
